# ASSOCIATIONS BETWEEN MEASURES OF SLEEP QUALITY, PHYSICAL ACTIVITY, ANXIETY, AND STRESS AMONG ADULTS

**DOI:** 10.1093/geroni/igac059.2323

**Published:** 2022-12-20

**Authors:** Sally Paulson, Michelle Gray, Anthony Campitelli, Joshua Gills, Megan Jones, Erica Madero, Jennifer Myers, Jordan Glenn

**Affiliations:** St. Elizabeth Healthcare, Cincinnati, Ohio, United States; University of Arkansas, Fayetteville, Arkansas, United States; University of Arkansas, Fayetteville, Arkansas, United States; University of Arkansas, Fayetteville, Arkansas, United States; University of Arkansas, Fayetteville, Arkansas, United States; Neurotrack Technologies, Redwood City, California, United States; Neurotrack Technologies, Redwood City, California, United States; Neurotrack Technologies, Redwood City, California, United States

## Abstract

Good quality sleep is important for physical and mental well-being and plays a vital role in healthy aging. Additionally, sleep quality is influenced by multiple factors associated with aging, such as lifestyle, depression, and physical activity levels. Therefore, the purpose of this study was to explore the relationship between self-reported measures of physical activity levels, anxiety, stress, and sleep quality among adults aged 45-75 years. Adults (N = 204) completed the short form International Physical Activity Questionnaire (IPAQ), Generalized Anxiety Disorder 7-Item Scale (GAD-7), Perceived Stress Scale (PSS), and the Pittsburgh Sleep Quality Index (PSQI). Participants were classified as either good quality sleepers (GQS, n = 65, 67.7% female, M±SD: age: 61.7±8.9 yrs; mass: 82.8±16.7 kg, height: 169.6±10.2 cm, education: 17.3 yrs) or poor quality sleepers (PQS, n = 139, 77.7% female, M±SD: age: 61.9±8.0 yrs; mass: 83.1±16.2 kg, height: 166.4±8.5 cm, education: 18.0 yrs) using their PSQI global score. A score of ≥5 denoted PQS. Data were analyzed using descriptive statistics, Spearman correlation, and Chi-square (p ≤ .05). Sleep quality was negatively associated with the PSS (r = -.17, p = .02) and GAD-7 (r = -.19, p < .01). Adults reporting PQS presented with higher levels of perceived stress and generalized anxiety. The relation between sleep quality and IPAQ was significant (X2 = 8.47, p = .01). These results suggest there was a greater tendency for PQS to report lower levels of physical activity and higher levels of stress and generalized anxiety.

